# ‘I’m definitely more confident now’: a qualitative study exploring how GP trainees learn about dermatology

**DOI:** 10.1093/skinhd/vzaf043

**Published:** 2025-06-25

**Authors:** Claire Doyle, Anne-Marie Tobin, Tony Foley

**Affiliations:** Department of Dermatology, South Infirmary Victoria University Hospital, Co. Cork, Ireland; Department of Dermatology, Tallaght University Hospital, Dublin, Ireland; Department of General Practice, School of Medicine, University College Cork, Co. Cork, Ireland

## Abstract

**Background:**

Despite a high frequency of presentations of skin disease in primary care, research has demonstrated a paucity of dermatology teaching in the medical school curriculum and a variable level of postgraduate dermatology teaching in general practice training. While it is clear that general practitioner (GP) trainees feel they would benefit from additional dermatology teaching, there is a gap in the literature regarding the factors that influence their learning.

**Objectives:**

The aim of this study was to identify and examine factors that influence the dermatology learning of GP trainees.

**Methods:**

A qualitative study of GP trainees in their third and fourth year of their training scheme practising in Ireland was undertaken. Full ethical approval was obtained prior to commencement. Semi-structured interviews were conducted with participants via Microsoft Teams. Participants were sampled using a combination of purposive, convenience and snowball sampling. Interviews were audio-recorded and transcribed verbatim. Data were analysed inductively by employing thematic analysis.

**Results:**

Fourteen GP trainees from five GP training schemes in Ireland participated in the study. Emergent themes identified factors that influence the dermatology learning of GP trainees in Ireland. These factors include experiential learning, competing demands, the clinical learning environment and mixed emotions towards dermatology. These influences mainly had a positive effect on the dermatology learning of trainees but certain competing demands such as time and incongruent teaching were felt to hinder their learning.

**Conclusion:**

This study broadens our understanding of factors that influence the dermatology learning of GP trainees. Findings generated from this study may be utilized to improve the clinical learning environment and inform the dermatology curriculum of GP trainees.

## Teaser

This novel qualitative study examines the factors that influence the dermatology learning of GP trainees. These factors include experiential learning, competing demands, the clinical learning environment and mixed emotions towards dermatology. These influences mainly had a positive effect on the dermatology learning of trainees but certain competing demands such as time and incongruent teaching were felt to hinder their learning.

**What is already known about this topic?**

- It is clear that general practitioner (GP) trainees feel they would benefit from additional dermatology teaching
- There is gap in the literature regarding the factors that influence the dermatology learning of GP trainees.
- The aim of this study was to identify and examine factors that influence the dermatology learning of GP trainees

**What does this study add?**

- This study adds real world experiential evidence to the factors that influence the dermatology learning of GP trainees.
- A novel finding was that of incongruent teaching of dermatology during other placements can lead to the subject matter of dermatology being difficult to conceptualize.

Skin diseases affect almost one-third of the global population.^1^ There is a significant psychological impact of skin disease, with 85% indicating that psychosocial elements are an important aspect of living with skin disease.^2,3^ Whilst it is difficult to quantify the financial cost of skin disease, it is estimated that moderate to severe atopic dermatitis alone results in annual societal costs in Europe of 30 billion euros.^4^ The central role of general practitioners (GPs) in the care of patients with dermatological conditions is recognized, with a Scottish group demonstrating that approximately 10% of all consultations to GPs were for skin disease while the Irish experience reports that 79% of GPs see one dermatological presentation per day.^5,6^

GP trainees find skin disease challenging, with high levels of consultation advice from supervisors, trainee-generated learning goals and high rates of in-practice follow-up.^7,8^ Contributory factors include a lack of undergraduate dermatology teaching and limited exposure to dermatology at a postgraduate level.^8^ In one US study, less than 40% of GP trainees felt the dermatology component of their medical school curriculum was sufficient and that dermatology teaching was deficient compared with diseases such as asthma and diabetes.^8^ A UK group reported that students at 25% of medical schools did not receive any dermatology education.^9^ An Irish study demonstrated that 7% of GP trainees reported a complete absence of undergraduate dermatology teaching.^6^

The postgraduate dermatology education of GP trainees varies. An Irish study reported that 43% of GP trainees surveyed reported zero dermatology teaching in their training.^6^ In contrast, American GP trainees were required to have at least 60 h of dermatology teaching.^8^ Despite the variable amount of postgraduate dermatology education, GP trainees reported that a rotation in dermatology was the third most useful hospital rotation for their training.^10^ Furthermore, 71% of a group of GPs surveyed want post-graduate dermatology teaching – the majority suggest that clinical training during their GP trainee years followed by updates at regular intervals would be most efficacious.^11^

Moreover, there is a paucity of undergraduate dermatology teaching in the medical school curriculum, with the average medical student receiving only 10 h of dedicated dermatology teaching.^5,9,12,13^ The vast majority (95%) of Irish medical students surveyed felt that their teaching in dermatology was insufficient and 89% would like more dermatology teaching.^6^ Unsurprisingly, it has been demonstrated that GP trainees who had the experience of an undergraduate placement in dermatology feel better prepared for the diagnosis and treatment of common skin diseases.^8^

While it is clear that GP trainees feel they would benefit from additional dermatology teaching, there is a gap in the literature regarding the factors that influence the dermatology learning of GP trainees. Adult learning is influenced by various factors including motivation, prior knowledge, and life experiences, as explained by adult learning theory.^14^ The integration of experiential learning and real-world contexts further supports effective adult education.^15^ Factors that influence the general learning experience of GP trainees include trainer availability and support, peer support, dedicated teaching time, the balance of personal learning and service delivery, entrustment, modelling, and personal factors such as physical and psychological health and psychosocial stressors.^16–18^ This study aims to explore GP trainees’ perspectives on how they learn dermatology during their training.

## Methods

### Sampling and recruitment of participants

This research focuses on GP trainees in the third and fourth year of their training schemes as they are seeing and treating skin diseases. A qualitative methodology involving semi-structured interviews was chosen as it facilitates deeper understanding of individual factors that influence the dermatology learning of GP trainees. Ethical approval was received from the University College Cork Social Research Ethics Committee (log number SOM/SREC/2023/2112/1). Recruitment was done via a combination of purposive and snowball sampling. Initially, professional contacts of the lead researcher and the lead supervisor who met the inclusion criteria were approached individually. Subsequently, these participants disseminated details for the study amongst suitable peers who were asked to contact the lead researcher if they were interested in participating.

### Data collection

Semi-structured interviews were conducted using a predefined topic guide which was created based on an extensive literature review and established guidelines that were relevant to the undertaking of qualitative research.^19^ Prior to creation, topics were discussed by the research team (CD Dermatology Specialist Registrar and TF academic GP with experience in qualitative research) and revised multiple times. The interview guide was then piloted and revised following interviews with two GPs. Participants returned signed consent forms at least 24 h before interviews occurred. Verbal consent was acquired before interviews began. All interviews took place via Microsoft Teams. Demographics were attained using a data collection form immediately before interviews commenced. Interviews were audio recorded and transcribed verbatim. Interview transcripts were anonymized prior to analysis.

### Data analysis

Data was analysed inductively using thematic analysis.^20^ Thematic analysis is a six-step process that comprises data familiarization, generation of initial codes from the data, generating themes from the data, reviewing the themes, defining and naming themes, and generating the findings.^20^ All transcripts were uploaded to NVivo 14 software to facilitate analysis. During the initial coding process, codes were labelled as they were identified. Coding was undertaken by the lead researcher (C.D.). Initial codes were discussed between C.D. and T.F. Codes were then analysed and collated into potential themes and further reviewed, thereby generating a thematic map of the analysis. Continued analysis was undertaken to ensure the refinement and clarity of each theme. All transcripts were analysed and coded by each researcher using the principle of constant comparison.^21^ The researchers met on multiple occasions to refine themes and further develop subthemes. The authors adhered to the consolidated criteria for reporting qualitative research (COREQ) statement in reporting the findings of the study.^22^ Data sufficiency was achieved when no new insights from participants emerged. Thematic saturation appeared to have been reached when 11 interviews had been undertaken. A further three interviews were undertaken to confirm this, in line with the method described by Francis *et al.*^23^

## Results

Fourteen GP trainees took part in this study. Table 1 details participant demographics. Median interview duration was 30 min (range 24–43 min).

**Table 1:** Demographics of study participants

Variables	Frequency (*N*)	Frequency (%)
Gender		
Male	6	44
Female	8	56
Year of the training scheme		
3rd year	6	44
4th year	8	56
Location of the training scheme		
South	5	36
East	2	14
North East	1	8
North West	3	21
West	3	21
Previous experience working in dermatology before community practice		
Yes	5	36
No	9	64

For this study, the analysis focused on identifying and describing the various factors that influence how GP trainees learn about dermatological conditions. The four emergent themes identified include;

- Experiential learning
- Competing demands
- Clinical learning environments
- Mixed emotions towards dermatology

See Table 2 further details themes and subthemes.

**Table 2:** Presentation of themes and subthemes

Theme	Subthemes
(i) Experiential learning	Improving confidence from 3rd to 4th year of training scheme
	Visual nature of dermatology
	Improved confidence in recommending treatments
(ii) Competing demands	Time
	Incongruent teaching
	COVID-19 pandemic
	Devoting attention to emergency presentations over dermatology
(iii) Clinical learning environments	Trainer approachabilityTrainer interest in dermatologyDedicated teaching in the clinical learning environment


(iv) Mixed emotions towards dermatology	Enjoyment of dermatologyGaps in dermatology knowledgeMotivated nature of GP traineesSelf-directed nature of post-graduate teaching




GP, general practitioner.

### Experiential learning

A recurring theme that emerged in the interviews was the sentiment of participant improvement and increased levels of confidence with more clinical experience. This was something that was expressed by all interviewees. Experiential learning is used to refer to the concept of learning through lived experience.^24^ This is an established feature of postgraduate medical training.^24^

‘I’m definitely more confident now just by virtue of experience and seeing loads of dermatology.’ (Participant 14)

A particular area that emerged within experiential learning was the improvement in both participant knowledge and confidence in dermatology from their third year on the training scheme.

‘In third year, you’re kind of getting a feel for if you’re still having a feel for loads of other things you’re still trying to just get to grips with everything, then in fourth year you’ve come across things a few times and you are more switched on.’ (Participant 7)

Interviewees felt less fear regarding dermatological presentations as they progressed through their training scheme.

‘I’m sure you’d probably get a very different interview off me at the start of third year. I’d probably have been be much more afraid of skin and stuff.’ (Participant 6)

The unique visual nature of dermatology as a speciality emerged within the theme of experiential learning. Diagnoses in dermatology are frequently made based on pattern recognition without the need for further investigations. Participants expressed views that this unique aspect of the speciality meant that through seeing and experiencing presentations they would recognize presentations of the same condition in the future.

‘Yes, recently the GP partner brought me into his room to see that Christmas tree rash… what’s it called… pityriasis rosea. That was interesting as I had never seen it before but now that I have seen it, I will know to recognize it in the future. I think dermatology is all about pattern recognition and once you’ve seen something in clinical practice it is easier to recognize it in the future.’ (Participant 2)

As both clinical experience and as a consequence, knowledge improved participants were more aware of previously missed diagnoses.

‘I thought there were no patients (with HS) in my practice but then once I started seeing patients, I diagnosed 4 patients. I think if you just don’t know where to look at for, you don’t see it.’ (Participant 10)

In addition, as trainees engaged in more experiential learning they felt more confident in counselling patients regarding treatment.

‘I’ve been more confident with my advice around eczema and using topical steroids than I would have been. And you need to be confident in that. Yeah, but they’re scary (topical steroids) at the start because parents are scared by them and you don’t have courage to talk about them.’ (Participant 6)

### Competing demands

The theme of competing demands in this context references anything that interviewees felt that their dermatology learning was compromised by in some way. A subtheme that emerged from this was time dedicated to the subject matter of dermatology. This is especially pertinent to the area of GP training as time needs to be dedicated to a multitude of areas. The feeling of needing to balance dermatology learning against many other important specialities was echoed by many participants.

‘Every medical speciality has its place in general practice. So you need to sort of divide your time a little bit.’ (Participant 1)

A subtheme that became apparent within competing demands was that of incongruent teaching. In this context, this is used to reference dermatology teaching that occurs during the hospital placement years of the GP training scheme when interviewees were on rotations where they were unlikely to have encountered any dermatology. Many participants felt that this made dermatology as a subject difficult to conceptualize.

‘Dermatology teaching happening early in the scheme can mean that it is less relevant. Lectures on psoriasis when you are on psychiatry means it is harder to pay attention.’ (Participant 13)

However, not all participants agreed with this statement with others feeling that this aspect of teaching reminded them of the breadth of knowledge required in general practice.

‘Actually it breaks up the monotony a little bit and it reminds you that you’re doing GP as well because you can kind of really lose the light at the end of the tunnel when you’re in your hospital attachments.’ (Participant 5)

A unique influence on the current group of GP trainees was the of lack of face-to-face teaching during the COVID-19 pandemic. This is because some of their training occurred during this period.

‘The teaching was all online. I think it would be nice to get some in-person teaching as well, you know because I think it could be helpful to just ask people in person and get that kind of what I want, feedback that you don’t get with Zoom.’ (Participant 10)

It was also expressed that phone consultations mean less opportunity to see and examine patients and therefore less time to add to experiential learning.

‘And then like, say, working in 2020, you know, like all the clinics kind of not happening and a lot of phone consultations, I suppose, just we’re seeing as many face-to-face presentations.’ (Participant 10)

Some participants expressed a desire to have face-to-face exposure in dermatology.

‘I do think spending time in a clinic or on dermatology placements would be so helpful to learn from what is seen in clinic.’ (Participant 3)

Some participants felt a barrier to their dermatology learning was the importance of devoting attention to emergency presentations over dermatological presentations. This was particularly felt by those who had gone straight from their intern year onto the GP training scheme.

‘There can be other things that would take priority because you’re like, OK, if I’m not up on my management of an acute asthma attack or a seizure or something when I’m going out in the first three months of third year.’ (Participant 12)

This contrasts with the opinions of other participants who felt that dermatology should be a mandatory rotation during GP training.

‘So I think that placement in dermatology should be mandatory like the way that our placement in paediatrics is mandatory. I think that it should be a mandatory placement because it is a huge part of what we see and it’s such a subspecialized area in the hospital that even though you may be working in adult medicine etcetera, etcetera, you may not see it.’ (Participant 4)

### Clinical learning environment

The clinical learning environment (CLE) refers to the clinical setting where health professions students undertake clinical placements as part of their education.^24^ In the context of this study, this is the GP practice where they are working as part of their training schemes. A frequently mentioned aspect of the CLE that was mentioned was the trainer of the interviewee and how approachable the interviewee felt they were.

‘I just literally go, would you mind reviewing a rash with me and he comes into me pretty quickly.’ (Participant 11)

Some participants had the experience of trainers dismissing them when they approached them with a dermatology query with a result that said participant feels they cannot discuss dermatology cases with their trainer.

‘Recently I asked my trainer about a rash and he just said I should have known that and didn’t explain it to me. So I’m not really comfortable asking him now about any rashes. I suppose it’s that thing of having someone who’s training you, someone approachable that you can ask, because it’s mostly so much exposure.’ (Participant 10)

Furthermore, an additional factor that influenced the dermatology learning of GP trainees was that of training interest in dermatology. Those who worked with a trainer with an interest in dermatology felt that they benefited from working with trainers who had an interest in dermatology.

‘Most of my learning and teaching in dermatology has been through the GP right here.’ (Participant 1)

‘I think your own trainer’s knowledge in the area. You know, if they’re not interested in dermatology, they don’t see a lot of it. You’ll have many more referrals onwards, so I think that that’s a big aspect of it to GP trainees as well.’ (Participant 4)

Other participants expressed that their current trainer was not as interested in dermatology as other areas which they felt impacted the learning they had in that particular CLE.

‘My trainer would have said that he’s not the strongest either on dermatology. Like any area of medicine with your trainer, because people have their own sort of background and like my trainer last year would have been pretty good. So it’s the luck of the draw a little bit.’ (Participant 5)

Similarly, dedicated teaching in the subject of dermatology in the CLE was found to have a positive impact on the dermatology learning of GP trainees.

‘To be honest the majority of my teaching has been on the job as I have had excellent dermatology tutorials with my trainer in my practice.’ (Participant 4)

‘I think just through exposure and through tutorials with my trainer now I’m a bit more comfortable and now wouldn’t spontaneously volunteer that dermatology is one of my biggest weak points whereas I had felt it was a big area of weakness last year coming from the emergency department.’ (Participant 1)

### Mixed emotions towards dermatology

The feelings of participants towards dermatology influenced their dermatology learning. Many participants enjoyed dermatology as an area and therefore were motivated to seek extra dedicated dermatology teaching.

‘I’m doing the dermatology diploma UCD at the moment and because I like the area, we see a huge amount of it, but I just felt that I hadn’t got enough training in it.’ (Participant 4)

Another participant who enjoyed dermatology created an anonymized logbook of dermatology presentations to reflect on diagnoses and presentations.

‘I started to keep the pictures definitely anonymous picture just for the lesions and what diagnosis I have made and I was comparing it to with the response what I’m doing and if I’m sending them for samples. A log book of sorts.’ (Participant 13)

Not all participants shared positive views of dermatology, which was in contrast to the overall positive views of participants.

‘To be honest it isn’t something I particularly enjoy when a person comes in with a skin issue.’ (Participant 3)

Furthermore, certain participants expressed the feelings that there were significant gaps in their dermatology knowledge. Participants identified these gaps and then sought out dermatology teaching based on said gaps.

‘My trainer saw perioral dermatitis for me after the case we did a tutorial on the management of perioral dermatitis which was really good. It was especially helpful after seeing the presentation.’ (Participant 9)

Other participants sought additional learning experiences as they felt their dermatology knowledge was somewhat lacking.

‘I would have done the ICGP derm course to try and kind of get a bit better. And then I’ve bought a couple of books. I bought Dave Buckley’s book just to have a flick through and Amanda Oakley the DERMNET ladies book as well, just to kind of improve.’ (Participant 6)

The motivated nature of GP trainees to engage in further learning was another subtheme that emerged regardless of their feelings towards dermatology.

‘I think like the GP trainees are generally very keen to access any kind of teaching, really that’ll help us cause I think very we feel like we were one minute we are SHOs and then the next minute was just us and the patient in the room and we’re making all the decisions all of a sudden.’ (Participant 11)

Some participants struggled with the nature of self-directed learning which is a feature of postgraduate medical training.

It’s very difficult. I suppose with a lot of things, like the membership exams, a lot of them is self-directed and you do kind of have to just study a lot of the stuff which is the same for GP training.’ (Participant 3)

## Discussion

### Summary of key findings

This novel interview study is the first to focus on the various factors that influence the dermatology learning of GP trainees. The overall learning experiences of GP trainees are influenced by a myriad of factors including trainer availability, willingness of trainer to engage with learning opportunities, peer support, learning portfolios, assignments and examinations, dedicated teaching, the balance of personal learning and service delivery, entrustment, modelling, and personal factors such as physical and psychological health and psychosocial stressors.^16–18,25^ GP trainees interviewed as part of this study identified factors that both augmented and hindered their dermatology learning experience.

A key finding from the study was the unique competing commitments that are faced by GP registrars in training. These competing commitments include incongruent teaching and balancing learning time across all areas in general practice. In the context of this study, incongruent teaching refers to dermatology teaching when a trainee was working in a speciality with little or no exposure to dermatology. This was felt to be a barrier to dermatology learning as trainees felt that dermatological diseases were difficult to conceptualize and therefore made engagement with the topic more challenging. This was particularly marked in the years of training prior to community practice. Dedicating adequate time to the various specialities that present to general practice was another competing commitment reported by participants. While it was recognized that skin disease is a frequent issue that presents in general practice, participants felt that other areas particularly emergency presentations required more of their focus. Participants reported that their trainer’s interest in dermatology and the accessibility of their trainer had both a positive and negative impact on their dermatology learning depending on the individual context.

### Comparison with existing literature

To the best of our knowledge, this is the first study that examines the influences on the dermatology learning of GP registrars. Interviewees reported that incongruent teaching acted as a barrier to their dermatology learning. A qualitative study reports that GP trainees would prefer to have GP placement in parallel to hospital placement to contextualize their learning to the GP setting, which mirrors the concept of incongruent teaching impacting the conceptualization of a subject matter.^17^ Echoing this, a Swedish qualitative study demonstrated the importance of contextualizing learning for medical students on placement, with students finding it challenging to join theoretical knowledge with what they see in clinical practice.^26^ The concept of incongruent teaching in the area of postgraduate training appears to be lacking in the literature, which may indicate that this is an accepted aspect of medical training. Self-directed learning and self-regulation are accepted features of postgraduate medical training.^27^ Often postgraduate medical learners are required to rely on intrinsic motivation and the teaching received may not be related to their day-to-day practice. Incongruent teaching may also be a barrier to teaching and learning in other aspects of the GP trainee curriculum.

Lack of time because of competing commitments was identified by participants as a factor that negatively impacted their dermatology learning. This finding echoes previous research which found that time is the most significant and common barrier for GP trainees in the completion of workplace-based assessments, thereby emphasizing the significant time pressure that GP trainees are under, despite being in a position of training.^28^ It is widely acknowledged that both lack of time and the balance between learning and service provision are ubiquitous features of postgraduate medical training.^28^ Suggestions for clinical teachers to overcome this barrier include identifying the needs of the learner, tailoring their teaching based on these needs and providing prompt feedback.^29^ Furthermore, it has been demonstrated that even opportunistic, informal teaching encounters can benefit the learning of trainees.^30^ This finding highlights challenges for GP trainees and GP trainers in time-poor environments, prompting the need to promote flexible teaching strategies that could be implemented to improve trainee learning.

Interviewees reported that both their trainer’s view towards dermatology and the approachability and accessibility of their trainer influenced their dermatology learning. This finding was viewed through both a positive and negative lens, and this was dependent on the behaviour of the trainer. In line with our findings, the wider literature supports the influence of the GP trainer on the learning experience of the GP trainee. The role of the GP trainer in the evolution of the GP trainee to an independent GP is vital.^31^ It is suggested that, to minimize learning barriers of trainees GP trainers should amongst other things ‘provide a supported learning environment’.^31^ Trainer accessibility is a recognized factor required for the development of a trainee’s trust in their trainer which is important for the trainee on the road to competent and independent medical practice.^32^ The literature also affirms that the overall confidence of GP trainees was increased when they had a trainer who helped them identify learning needs and provided dedicated teaching in a particular area.^33^ This mirrors the findings of our study where participants who had worked with a GP with an interest in dermatology or one who organized dedicated teaching sessions on dermatological presentations felt this had benefited and influenced their dermatological learning.

### Strengths and limitations

This study used a qualitative design which was chosen to capture the breadth and depth of the feelings and experiences of GP trainees. The purposive sampling strategy employed by the research team led to a diverse participant group in terms of gender, year of training, training hub, practice location and prior experience in dermatology. Furthermore, our research team brought expertise both in dermatology and in general practice, thereby bringing a real-world lens to the research undertaken.

Limitations of the study include the possibility of social desirability bias. The primary researcher and interviewer is a dermatology specialist registrar. Arising from this position, GP trainees may have framed their answers about dermatology in a positive light. To reduce this potential bias, the interviewer assured participants before commencing the interviews that the questions would focus on their experience and opinions rather than particular knowledge. Another potential limitation was that of volunteer bias.^34^ Participation was voluntary, meaning that those who are willing to engage with the project represent a certain group of GP trainees. The participants may be more confident and experienced in dermatology than other GP trainees which may motivate them to partake in dermatology-focused research.

### Implications for research and practice

The implications of this work for research should be informed by all relevant stakeholders, patients and clinicians. It is important to develop bespoke educational interventions that meet the dermatological learning needs of GPs of the future. For instance, a systematic review of the literature on the educational interventions that have proved beneficial for GP trainees is warranted. In addition, triangulating perspectives of patients, GPs and dermatologists, a Delphi Consensus study could help to inform the design of an appropriate and relevant dermatology curriculum for GP trainees.

Regarding implications for clinical practice, the findings from this study indicate that GP trainees feel that more dermatology teaching, at both undergraduate and postgraduate level, would be beneficial for their clinical learning. Therefore, it is important that curriculum developers in medical schools and in GP training are informed of this educational need and include the diagnosis and management of common dermatological conditions in their curricula. Practical strategies for educational interventions in the future could include the promotion of opportunistic, informal teaching sessions by GP trainers as well as more formal, structured teaching opportunities and clinical experience, facilitated by local dermatologists or GPs with an extended role in dermatology. Online educational resources containing content tailored to the needs of GPs could be used such as the Primary Care Dermatology Society^35^ website or DermNet.^36^ GP training schemes should offer some dermatology rotations as part of GP training or, at least, the ability to attend dermatology clinics during dedicated, protected teaching time.

As clinicians and educators, our primary aim should be to meet the needs of our patients, by ensuring that the doctors of the future have the requisite knowledge and skills to care for them. The findings generated from this study can be utilized to better understand the factors that influence dermatological learning, thereby improving the dermatological educational experience, skills and confidence of GP trainees.

## Data Availability

The data underlying this article will be shared on reasonable request to the corresponding author.
